# Single-cell profiling defines the cellular landscape of the urinary bladder: a scoping review

**DOI:** 10.1186/s40001-025-03750-6

**Published:** 2026-01-05

**Authors:** Eugene Padi, Alexander Guldmann Clausen, Anastasia Buch Kjeldgaard, Mahboobeh Amoushahi, Clara Ibel Chamorro, Magdalena Fossum

**Affiliations:** 1 Laboratory of Tissue Engineering, Rigshospitalet, Faculty of Health and Medical Sciences, University of Copenhagen, Copenhagen, Denmark; 2 Division of Pediatric Surgery, Department of Surgery and Transplantation, Copenhagen University Hospital, Rigshospitalet, Copenhagen, Denmark; 3 Laboratory of Tissue Engineering, Department of Women’s and Children’s Health, Center of Molecular Medicine, Karolinska Institutet, Stockholm, Sweden

**Keywords:** ScRNA-seq, Transcriptomics, Bladder, Unsupervised clustering, Differentially expressed genes

## Abstract

**Background:**

The urinary bladder contains a heterogeneous cell population. Multiple studies utilizing single-cell RNA sequencing techniques have uncovered the complex transcriptomic profile of cells in the healthy bladder.

**Objective:**

The present study aims to map existing evidence on the use of single-cell RNA sequencing to assess cellular heterogeneity in the healthy urinary bladder and to identify gaps in the literature that could guide future research.

**Design:**

We searched four online databases, supplemented with manual searches, to identify relevant studies that characterized various cell types of the bladder at single-cell resolution. Studies that did not meet the predefined inclusion criteria were excluded based on a protocol drafted a priori. We included only studies published in English.

**Results:**

Twelve studies met the inclusion criteria, investigating various subtypes of cells: urothelial, interstitial, smooth muscle, immune, endothelial, mesothelial, and neural. A landmark study differentiating mural cells from fibroblasts was also presented. Another study compared the use of single-cell with single-nuclei RNA sequencing to assess the utility of the latter for cell identification. We further discussed the gaps and requirements necessary to implement single-cell RNA sequencing in a clinical setting.

**Conclusion:**

The combined results highlight several rare and newly identified cell types in different compartments of the urinary bladder. Data integration and validation across multiple modalities are critical steps forward to resolve the cellular complexity of the urinary bladder.

**Supplementary Information:**

The online version contains supplementary material available at 10.1186/s40001-025-03750-6.

## Introduction

The urinary bladder harbors a diverse array of cell types, including urothelial, fibroblast, immune, and smooth muscle cells. While each cell type holds unique characteristics, they interact to ensure proper storage and voluntary expulsion of urine. For instance, proximity to urine requires tight protective barriers of urothelial cells that shield the underlying tissues from toxic substances in urine and defend against invading pathogens. This primary role of urothelial cells is managed partly by umbrella cells that form a mucin and glycosaminoglycan barrier at the apical surface layer of the urothelium [1]. Other cells, such as fibroblasts, constitute part of the interstitial cell layer, where they secrete factors that support the overlying urothelial cells, thereby maintaining tissue repair and homeostasis. Also present in the multicellular niche are resident immune cells that scout various regions of the bladder for potential invasive pathogens. Mechano-sensory cells are also found in the urothelium, sensing changes in the extracellular environment and relaying signals to the afferent nerves and smooth muscle cells, resulting in urinary bladder distension and contraction [1]. Collectively, distinct cells populate various regions of the urinary bladder to support its physiology as a low-pressure reservoir.

Knowledge regarding these unique cell types and their functions in the urinary bladder has previously been propelled by anatomical and histological studies capturing the expression of a subset of marker genes or proteins [2–4]. While such investigations provide insights into bladder morphology, cellular compartments across the tissue are often overlooked. Unsurprisingly, urothelial cells in the bladder are morphologically identical and share a similar basic histological structure with those across the urinary tract [5]. However, various subpopulations have been identified across the different compartments [6–8]. Thus, histologically identical cells appear to have distinct transcriptional signatures and functions.

**Fig. 1:**
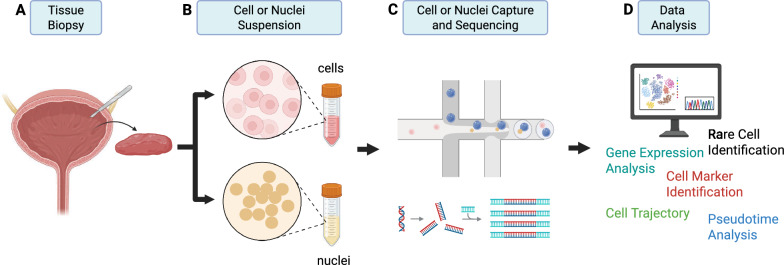
Workflow of a single-cell or single-nuclei RNA sequencing of the urinary bladder by droplet-based method. **A**, **B** A piece of urinary bladder tissue is resected and dissociated into a single cell or nuclei suspension using mechanical and enzymatic digestion. **C** The resulting cell or nuclei suspension is then passed through a microfluidic device with required reagents, which randomly encapsulate the individual cells or nuclei into droplets. The encapsulated cells or nuclei are subjected to sequencing. **D** Data analysis is conducted to identify gene expression, cellular heterogeneity, rare cells, cell trajectory, etc. Created in https://BioRender.com

To discriminate between cell types, recent advances in single-cell technologies are unravelling novel and rare cell populations in the urinary bladder [6, 8, 9]. Single-cell RNA sequencing (scRNA-seq) is a formidable tool that enables high-throughput transcriptomic profiling of individual cells using next-generation sequencing [10] (Fig. 1). With this approach, several subsets of fibroblasts have been identified in the suburothelium and intramuscular layers with implications for specific bladder functions and diseases [11, 12].

Discovering distinct cell types using single-cell technologies raises a list of unanswered questions in bladder physiology. What contributes to cellular heterogeneity in the urinary bladder? How susceptible are bladder cells to changes from external cues? Which subsets of cells could be targeted for therapeutic benefits and at which state should the cells be targeted? Various factors including sex, age, and disease all influence cellular diversity. Studies have postulated a sexually dimorphic bladder wherein the epithelial and mesenchymal cells are distinct between male and female mouse bladders [13, 14]. Likewise, age happens to alter the cellular landscape of the bladder; thus contributing to the presence of a unique repertoire of fibroblasts, immune cells, and other cell types [11, 13]. Although the physiology of the urinary bladder is far from being fully characterized, the diseased state is equally challenging. Bladder cancer is among the most complex and aggressive forms of cancer owing to the contribution of multiple cells and genomic heterogeneity [15]. As a result, the clinical prognosis of tumors of the bladder remains unpredictable.

Our understanding of the human bladder at single-cell resolution is still largely incomplete. Therefore, defining the cellular diversity is essential for understanding the physiology and diseases associated with the urinary bladder. This scoping review aims to encapsulate existing evidence from single-cell or single-nuclei RNA sequencing (sc/snRNA-seq) studies that investigated cellular diversity in the healthy urinary bladder. We also identify gaps in the literature that could guide future research in this field. Using the Population–Concept–Context (PCC) framework, this scoping review focuses on (1) Population: cells derived from human and other mammalian urinary bladder tissues; (2) Concept: cellular heterogeneity, including distinct cell types and transcriptional profiles as described by sc/snRNA-seq; (3) Context: out of scope due to the nature of the studies. Hence, the scoping review addresses the following research question: In the healthy urinary bladder, what cell populations, states, and transcriptional signatures have been reported using sc/snRNA-seq?

## Methods

### Protocol and registration

A protocol was initially drafted following the Joanna Briggs Institute template for protocol registration, and subsequently revised by the co-authors. The final protocol was registered with the Open Science Framework on December 4, 2024 (https://osf.io/gju6k) [16]. Deviation: The protocol was drafted with a focus on both the urinary bladder and the urethra; however, due to the space limitation and current structure of the article, we covered only the urinary bladder.

### Eligibility criteria

A comprehensive literature search was performed to identify studies that met the inclusion criteria: studies reporting original sc/snRNA-seq of the healthy mammalian urinary bladder. Exclusion criteria included studies using in vitro models, genetically modified animal models, tissues from patients with diseases involving the lower urinary tract (with a focus on the disease), disease models, pharmacological interventions, proteomics, and epigenetics (e.g., microRNA). Studies that exclusively used other techniques such as microarray, RT-qPCR, histology, RNA (bulk) sequencing, and in situ hybridization were also excluded.

### Information sources and search

We searched EMBASE, PubMed (MEDLINE), Scopus, and Web of Science for original articles that met the inclusion criteria. The last search for articles was conducted on February 17, 2025. The search strategy was drafted and revised with support from an information specialist at the University of Copenhagen Library. The search strategy involved a mix of controlled vocabulary (e.g., MeSH) and keywords (Supplementary Table 1). The search string was restricted to studies that involved non-cancerous tissues and no other restrictions (e.g., date, species, etc.). Additional studies, including one preprint, were included from the reference lists of relevant articles and targeted Google searches, in which we applied keywords derived from our inclusion criteria. Details of the search strategy are provided in Supplementary Table 1. The final search results were exported to Covidence for screening.

### Selection of sources of evidence

The screening process was carried out independently by two authors (AGC and EP) using Covidence. All titles and abstracts were screened for eligibility based on the specified inclusion and exclusion criteria. Full-text review and data extraction were conducted following the initial screening. All articles, including those from additional sources, underwent the same review and assessment process. Disagreements regarding study selection and data extraction were addressed through a structured resolution process. Discrepancies between reviewers were identified and discussed in Covidence. If consensus could not be reached, a third reviewer acted as an arbitrator to ensure a fair and consistent study selection process.

### Data charting process

Two reviewers (EP and AGC) developed a data charting form to identify which key variables to extract from the included studies. The reviewers discussed and revised the template prior to data extraction. One author extracted the data and co-authors verified the accuracy of the data. Discrepancies were resolved through discussion.

### Data items

The extracted data included article characteristics (e.g., title, author, year, and key findings), experimental design (e.g., source of tissue and number of tissues), and gene expression profile (e.g., number of reported cell clusters, gene signature, and data repository).

### Synthesis of results

The studies were grouped based on the type of cells found in the urinary bladder (e.g., urothelial cells, interstitial cells, etc.), along with the study characteristics, synthesis of gene signatures, and key findings related to the bladder. When a study analyzed multiple cell types, we separated the narrative based on each individual cell population. Therefore, a study could be found in multiple sections, but each chapter presented findings of a specific cell population from that study.

## Results

**Fig. 2:**
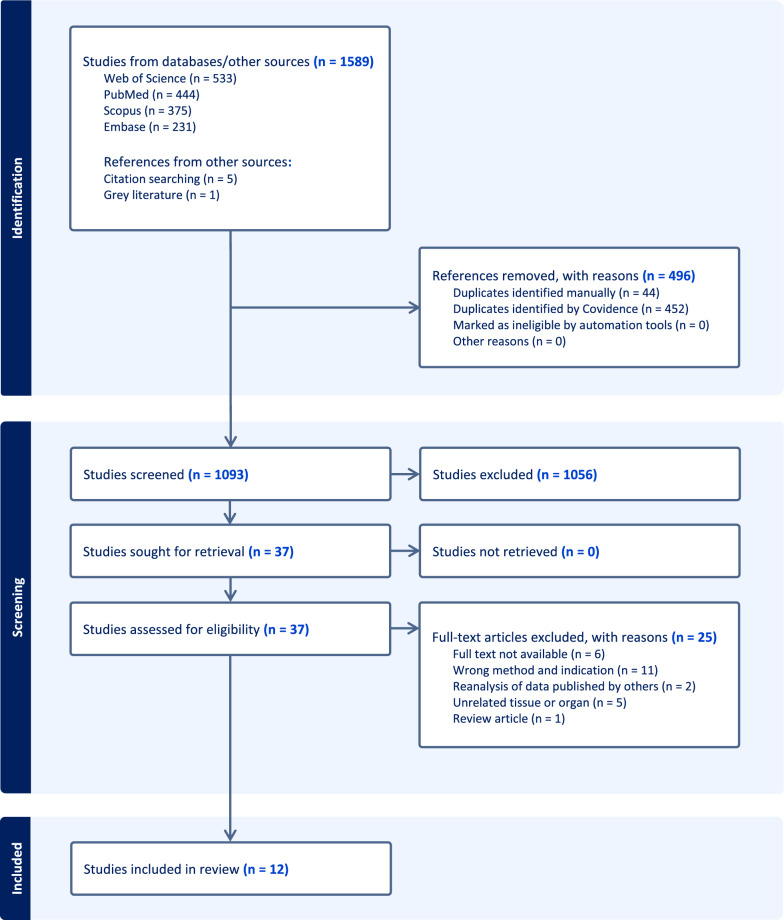
PRISMA flowchart. A total of 1589 articles were identified from the initial search through EMBASE, PubMed (MEDLINE), Scopus, Web of Science, and others. After assessing abstracts and conducting full-text screening, we selected 12 eligible studies for this review

The literature search generated 1589 articles of which 12 were considered eligible for this review. The search strategy, screening, and identification were summarized in the PRISMA flow diagram (Fig. 2). We excluded two studies that re-analyzed published datasets. Of the included studies, most analyzed the healthy urinary bladder from animals (e.g., mice or rats). Studies that used human tissues acquired bladder samples from deceased patients or patients undergoing cystectomy due to malignancy. The cystectomy samples were resected at a distance from the malignancy and deemed histologically healthy. One study investigated the cellular landscape of the aging bladder. Below, we have subdivided the studies based on cell types and further presented studies that viewed the cells of the urinary bladder in comparison to others or investigated different methodologies of scRNA-seq technology. Characteristics of all studies are summarized in Table 1.

**Table 1:** Summary of study characteristics

Authors, year	Tissue source	Sex (biological replicates)	Bladder cell types identified (n)	Key findings	Single-cell isolation/Platform
Yu et al 2019 [6]	Patients with bladder cancer Wild-type C57BL/6J mice	Human: Female (*n* = 1) Male (*n* = 2) Mouse: Female (*n* = 5) Male (*n* = 5)	Human: 16 Mouse: 15	The study suggested that *ADRA2A*^+^/*HRH2*^+^ interstitial cells are implicated in nerve conduction and allergic reactions while *TNNT1*^+^ epithelial cells may play a role in bladder emptying. Human and mouse bladder cells shared a high number of homologous genes	Microfluidic droplets/10x Genomics
Zhao et al. 2023 [17]	Patients with bladder cancer Sprague–Dawley rats	Human: Female (*n* = 1) Male (*n* = 1) Rats: Female (*n* = 1) Male (n = 1)	Human: 12 Rat: 8	Two types of interstitial cells were characterized in the subepithelial lamina propria, and between muscle bundles	Microfluidic droplets/10x Genomics
Li et al. 2021 [9]	Wild-type C57BL/6J mice	Female (*n* = 5)	8	The study suggested that *Plxna4*^+^ urothelial cells may be involved in host response to infection and wound healing *Aspm*^+^ basal cells proliferated after injury	Microfluidic droplets/10x Genomics
Baker et al. 2021 [11]	Wild-type C57BL/6J mice	Female (*n* = 13) Male (*n* = 16)	25	Three novel fibroblast clusters: *Car3*^+^ suburothelial fibroblasts, *Npy1r*^+^ lamina propria fibroblasts, and *Penk*^+^ detrusor muscle fibroblasts snRNA-seq favored the capture of a broad transcriptional profile of detrusor-specific smooth muscle cell population	Microfluidic droplets/10x Genomics
Muhl et al. 2020 [8]	Wild-type C57BL/6J mice. Multiple mice strains including *Acta2*^*GFP*^ reporter mice	Male (*n* = 24)	11	Mural cells were less heterogeneous than fibroblasts *Tnc*^+^*Cd34*^*−*^ fibroblasts resided in the suburothelium while *Tnc*^*−*^*Cd34*^+^ fibroblasts were present beneath the bladder mucosa	FACS*/SmartSeq2
Ligon et al. 2020 [13]	Wild-type C57BL/6J mice	Female (not specified)	21	Aged bladders had increased population of B cells and T cells Bladder tertiary lymphoid structures colonized the aged bladder	Microfluidic droplets/10x Genomics
Han et al. 2018 [24]	Wild-type C57BL/6J mice. Cell lines	Male (*n* = 1)	16	Two stromal cell populations, one highly expressing *Dpt* and the other expressing *Car3*, were specific to the mouse bladder Bladder-related macrophages shared similar transcriptomes with those of the testis, pancreas and mammary gland	Agarose microarray/Microwell-seq
Han et al. 2020 [21]	Healthy fetus and adult human Cell lines	Female (*n* = 1) Male (*n* = 1)	Female: 12 Male: 8	Bladder-specific endothelial cells expressed *HLA-DPA1*, *HLA-DRA*, and *PECAM1* Endothelial cells, *CXCL*^+^ epithelial cells, and stromal cells expressed immune-related genes involved in tissue-specific immunity	Agarose microarray/Microwell-seq
He et al. 2020 [25]	Healthy adult human	Male (*n* = 1)	19	FibSmo stromal cells were detected mainly in the submucosa of the bladder Epithelial cells of the bladder shared similar TFs to those of non-digestive organs (i.e., trachea)	Microfluidic droplets/10x Genomics
The Tabula Muris Consortium 2018 [26]	Wild-type C57BL/6J mice	Female (*n* = 3)^∆^ Male (*n* = 4)	Droplet: 4 FACS: 2	Biases between FACS and droplet-based methods were confirmed, highlighting the loss of minor cell populations in the FACS-bladder datasets Transcriptional factors determined cell identity, which could potentially guide the generation of experimental protocols	FACS/SmartSeq2 and Microfluidic droplets/10x Genomics
The Tabula Muris Consortium 2020 [27]	Wild-type C57BL/6J mice	Female (*n* = 5)^∇^ Male (*n* = 16)	Droplet: 4 FACS: 2	Cellular composition and gene expression varied with age among which the bladder showed increased changes in urothelial and mesenchymal cells	FACS/SmartSeq2 and Microfluidic droplets/10x Genomics
Santo et al. 2025 [12]	Adult human	Male (*n* = 1)	14	Differences in cell type detection between scRNA-seq and snRNA-seq Non-coding RNAs dominated single-nuclei datasets snRNA-seq provided generic biological processes	Microfluidic droplets/10x Genomics

^*^FACS fluorescence-activated cell sorting

∆The bladder was excised from 3 females and 3 males with FACS and 1 female and 2 males with microfluidic droplets

∇The bladder was excised from 4 females and 9 males with FACS and 1 female and 7 males with microfluidic droplets

### Urothelial cells

Molecular features of urothelial cell populations have been discovered through scRNA-seq studies. Below, we have provided a synthesis of four studies that defined the subpopulations of urothelial cells.

Yu et al. conducted scRNA-seq analysis on three human bladder samples collected from patients undergoing radical or partial cystectomy (median age: 47 years) [6]. The authors identified six urothelial cell populations assigned as basal cells 1, basal cells 2, intermediate cells 1, intermediate cells 2, *TNNT1*^+^ epithelial cells, and umbrella cells. The study uncovered a novel type of urothelial cell that shared epithelial and muscle characteristics as denoted by the co-expression of epithelial (e.g., *KRT18* and *KRT19*) and skeletal muscle (e.g., *TNNT1*) genes. On multiple independent bladder samples from human, mouse, and rat, *TNNT1*^+^ epithelial cells stained positive in the umbrella and intermediate cell layers. Moreover, the study included bladder samples from mice, which were clustered into five distinct urothelial populations: basal cells 1, basal cells 2, intermediate cells, mixed epithelial cells, and umbrella cells. When comparing the human and mouse datasets, Yu et al. showed that mouse urothelial cells closely resembled human urothelial cells, albeit with few disparities.

In another study, Zhao et al. identified 12 major cell types in humans and eight in rats [17]. The characterization of these bladder cells was partially based on the expression of CD markers, which were further validated using a publicly available mouse dataset. Urothelial cells from both humans and rats were enriched in CD-associated genes (e.g., *CD326 (EPCAM)*, *CD138*, *CD358*, etc.). In addition, epithelial cells across all species (human, rat, and mouse) expressed uroplakin (e.g., *UPK3A* and *UPK1A*) and cytokeratin (e.g., *KRT19*) genes.

Similarly, Li and colleagues identified eight distinct subpopulations of urothelial cells in the mouse bladder following an unsupervised clustering analysis [9]. The subtypes of urothelial cells were termed basal cell 1, basal cell 2, basal cell 3, basal to superficial cell, intermediate cell, superficial cell 1, superficial cell 2, and *Plxna4*^+^ urothelial cell. Basal cell 1 represented cells that expressed canonical basal cell markers such as KRT5, SHH, and p63. Like basal cell 1, basal cell 2 and basal cell 3 also expressed similar canonical markers; however, basal cell 2 showed additional enrichment for DNA helicase genes (e.g., *Mcm2)*, whereas basal cell 3 expressed cell cycling regulatory genes (e.g., *Ccnb1*, *Hmmr*, and *Plk1*). Moreover, basal cell 3 expressed tricarboxylic acid cycle regulating genes, indicating the metabolic characteristics of this subcluster. In contrast, basal to superficial cell defined a transitional cell state from basal to superficial cells expressing both basal and superficial markers. The basal cell clusters also revealed the expression of a resident stem cell/progenitor marker, ASPM, which the authors validated in an injured model where the number of *Aspm*^+^ basal cells increased upon injury. *Aspm*^+^ cells were therefore denoted the progenitors/stem cells of the urothelium, as these subpopulations rapidly regenerated the injured urothelium. Other urothelial cells, including superficial cells, were further subdivided based on their expression of specific genes, such as *Cdh1* and *Tjp1*, which encode tight junction proteins, as well as the transcription factors *Foxa1* and *Grhl3*. Remarkably, the authors also identified a novel cell population of superficial cells, which they termed Plexin A4 (*Plxna4*^+^) urothelial cells. *Plxna4*^+^ urothelial cells also expressed the uroplakin gene, *Upk3b*, but not *Krt20*, further indicating that this subpopulation originates from the superficial layer (Supplementary Table 2). Immunofluorescence staining of Plexin A4 cells confirmed their location to the apical layer of the human, rat, and mouse urothelium.

In a preprint, Baker et al. challenged the current methodology for preparing bladder tissues for scRNA-seq [11]. The study involved three different optimized protocols, including one where the mucosa and detrusor were mechanically separated and treated independently for sequencing. With the optimized protocol, the authors analyzed additional cell populations often lost in conventional dissociation protocols for scRNA-seq. Clustering of cells resulted in seven major cell clusters representing fibroblast, urothelial, immune, smooth muscle, endothelial, Schwann, and mesothelial cells. Further analysis of subpopulations from the major cell types revealed 25 transcriptionally distinct clusters. Of the urothelial cell population, clusters expressing *Krt5* were denoted basal, and those enriched in *Upk2* represented the luminal/umbrella cells. The study also investigated the differentiation dynamics of these urothelial cells through a lineage trajectory analysis to predict the transcriptional differentiation of each cell type. The analysis resulted in five transcriptionally distinct urothelial clusters classified as early basal (*Gsdmc2*), basal (*Trp63*), intermediate (*Hes1*), luminal (*Upk2*), and late luminal (*Prss27*).

### Interstitial cells: fibroblasts and myofibroblasts

Interstitial cells (ICs) represent fibroblasts, myofibroblasts, and other connective tissues that play critical roles in supporting the extracellular matrix (ECM), tissue repair, and regeneration. Fibroblasts and myofibroblasts are present in several body compartments with subtypes specific to a given tissue. In this section, we survey the different interstitial cells that have been reported in scRNA-seq studies of the urinary bladder.

The Yu et al. study identified five disparate interstitial cell populations: fibroblast 1, fibroblast 2, fibroblast 3, myofibroblast, and *ADRA2A*^+^ interstitial cells [6]. Specifically, the mouse bladder contained interstitial cell populations analogous to those found in the human bladder, with the exception of the *ADRA2A*^+^ interstitial cells, which were unique to humans. All interstitial cells expressed the canonical gene for vimentin (*VIM*). A combination of genes, including *S100A4*, *COL1A1*, and *COL3A1*, was used to further subclassify fibroblasts into fibroblast 1, fibroblast 2, and fibroblast 3. *ADRA2A*^+^ interstitial cells constituted a newly identified cluster that expressed *VIM* and *ADRA2A*, and additionally co-expressed *HRH2* and *AVPR1A* encoding the α2-adrenergic, histamine, and vasopressin receptors, respectively (Supplementary Table 2).

In a separate study, Zhao et al. reported two major types as fibroblasts and myofibroblasts [17]. Additional clustering of the fibroblast population resulted in three different subtypes grouped as fibroblast 1, fibroblast 2, and fibroblast 3; although, individual gene signatures of each cluster were not fully described. All fibroblasts expressed genes including *PTN*, *IGFBP6*, *PI16* (*CD364*) and *CD34* (Supplementary Table 2). Gene ontology enrichment analysis also revealed functional differences across different subgroups of fibroblasts. Moreover, the authors validated their data by staining human, mouse, and rat bladder tissues with anti-PI16, revealing that these fibroblasts were present underneath the urothelium, specifically in the lamina propria and intermuscular regions. The other class of interstitial cells, myofibroblast, were characterized by markers including STC1, PLAT, TNC and TRPA1. Immunohistochemistry staining with anti-TRPA1, a gene expressed in myofibroblast, revealed that myofibroblasts were localized to areas between the urothelium and detrusor regions in human bladder tissues. However, the staining was negative in the rat and mouse bladders suggesting differences in function of myofibroblasts across species.

Evidence of *CD34*^+^ fibroblasts was also reported by Baker et al. [11]. Three subclusters of fibroblasts were identified each defined by the genes *Car3*, *Npy1r,* and *Penk* (Table 1). Using spatial transcriptomics and multiplexed immunofluorescence, Baker et al. demonstrated the presence of each fibroblast subtype within the regions of the bladder. *Car3*^+^ fibroblasts were located directly underneath the urothelium; hence, the authors coined this cluster of cells suburothelial fibroblasts (suF). Suburothelial fibroblasts also expressed myofibroblast genes (e.g., *Acta2*) and collagens. In contrast, *Npy1r*^+^ fibroblasts were detected around the lamina propria and detrusor while *Penk*^+^ fibroblasts were located primarily in the detrusor. Due to their respective locations in the bladder, *Npy1r*^+^ fibroblasts were classified as lamina propria fibroblasts (lpF) while *Penk*^+^ fibroblasts were termed detrusor muscle fibroblasts (dmF). Moreover, Baker et al. demonstrated the ability of the lpF to transdifferentiate into either suF or dmF in cell culture. The authors subjected *CD34*^+^ sorted cells to ligands and inhibitors of the TGFB1 or PDGFBB pathways. After 6 days in culture, activation of the PDGFBB (and inhibition of TGFB1) pathway increased the expression of dmF-related genes (*Dlk1*, *Penk*, *Gpx3*) in the *CD34*^+^ mucosal cells. Expression of suF-related genes (e.g., *Acta2*, *Cxcl14*) were also decreased in this condition. On the contrary, activating the TGFB1 and inhibiting PDGFBB pathway resulted in the expression of suF-related genes; however, there was a slight increase of dmF genes. Thus, in the presence of external cues consisting of ligands and inhibitors of TGFB1 and PDGFBB, *CD34*^+^ mucosal cells (e.g., lpF) could transdifferentiate into other subsets of fibroblasts.

The last study to be discussed is that of Muhl et al. [8]. In this study, muscular organs from the adult mouse, including bladder, heart, skeletal muscle, and colon were subjected to single-cell sequencing. Unsupervised clustering analysis conducted on the single-cell data revealed 16 unique clusters of cells across all four organs, 12 of which were fibroblasts and 4 mural cells (i.e., pericytes and vascular smooth muscle cells). The clustering analysis showed a close relationship between fibroblasts and mural cells. However, fibroblasts were best described by a spectrum of gene signatures, including *Cd34*, *Pdgfra* and ECM genes such as *Col1a1*, *Col1a2*, *Col5a1*, and *Fbln1*. Interestingly, genes that are associated with ECM production and maintenance significantly defined the heterogeneity among the fibroblasts, suggesting that ECM structures are tailored specifically to the individual organ’s requirement and function. Moreover, the authors described two major types of bladder-associated fibroblasts reported to be either *Tnc*^+^*Cd34*^*−*^ or *Tnc*^*−*^*Cd34*^+^ (Table 1). Immunofluorescence staining of bladder samples revealed that *Tnc*^+^*Cd34*^*−*^ fibroblasts were localized just below the urothelium. Conversely, *Tnc*^*−*^*Cd34*^+^ fibroblasts were found at deeper regions in the bladder wall. These findings corroborate results from Baker et al. wherein *Cd34*^+^ fibroblasts (lpF) primarily resided in the lamina propria but extended into the detrusor [11]. Likewise, *Tnc*^+^*Cd34*^*−*^ fibroblasts may represent the sub urothelial fibroblasts defined by Baker et al. as *Car3*^+^ suburothelial fibroblast. In fact, Han et al. 2018 presented the full transcriptome from their analysis confirming that *Tnc*^+^*Cd34*^*−*^ cells also expressed *Car3*.

### Detrusor smooth muscle cells

The detrusor represents the main muscle component of the urinary bladder, consisting of mainly smooth muscle cells embedded in an extracellular matrix [18]. Covering the anatomical regions of the bladder (fundus, dome, and neck) are longitudinally and circularly arranged smooth muscle fibers. The question remains whether smooth muscle cells along these anatomical locations are genetically identical or represent a heterogeneous population of cells. Capturing the diversity of smooth muscle cells remains challenging because of the organization of muscle fibers maintained through strong intercellular interactions. Thus, analysis of smooth muscles is often under-represented in scRNA-seq datasets. While the detrusor represents a significant portion of the bladder wall, current scRNA-seq datasets report a limited number of smooth muscle cells (1.1%) compared to other bladder cells [6, 11].

To mitigate this under-representation, Baker et al. attempted to capture the full transcriptome of the detrusor smooth muscle [11]. Briefly, the authors separated the detrusor from the mucosa and subjected the detrusor to an optimized dissociation protocol involving collagenase and papain, a milder digestion enzyme. Thereafter, the dissociated cells were subjected to scRNA-seq. The authors also utilized the snRNA-seq method on a whole bladder, which captures the transcripts of nuclei and is known to recover cells that are difficult to isolate. By combining both approaches, the transcriptomic datasets were compared to a bulk RNA sequencing dataset of a mouse bladder. Unsurprisingly, the results from the optimized scRNA-seq and snRNA were on par with the data from the bulk RNA sequencing. A subset of smooth muscle cells that was previously absent in the conventional approach of scRNA-seq was present in the optimized protocol and snRNA-seq datasets. These clusters highly expressed smooth muscle genes, including *Myh11* and *Myocd*, substantially increasing the total smooth muscle cell number from 315 (traditional approach) to 1763. The detrusor-enriched datasets were further subdivided into three distinct clusters: vascular smooth muscle (*Tesc, Pln, Wtip*), pericyte (*Rgs5, Kcnj8, Pdgfrb*), and detrusor smooth muscle (*Actg2, Acta1*, and *Tnnt2*). A spatial representation using imaging mass cytometry also confirmed the expression of *Actg2* in the detrusor layer, as well as *Acta2* expressed broadly in both the detrusor layer and the surrounding vascular structures. Furthermore, the clusters of detrusor smooth muscle cells contained neuronal-like transcripts (e.g., *Rbfox3, Kcnf1, Rims1*), which the authors postulated to be associated with the detrusor and involved in bladder functions (see section on neurons).

Overall, identifying the diversity across smooth muscle cells will require an optimized approach to elucidate their unique transcripts. Transcripts from the detrusor smooth muscle cells represent genes that support both contractile and neuronal functions in the bladder.

### Immune cells

The mucosa barrier represents the first line of defense against pathogens and toxins by clearing bacteria through the secretion of cytokines and antimicrobial compounds. Under homeostasis, the urinary bladder is quiescent, but proliferates rapidly upon injury [19]. The immune cell population of the bladder is approximately 80% macrophages and dendritic cells with a minor fraction of T cells, NK cells, mast cells, monocytes, and eosinophils under physiological conditions [13]. Efforts to describe the various resident and recruited immune cells of the bladder are sparse because most scRNA-seq studies rely on the dissociation of the whole bladder. In this section, we highlight two studies that described the heterogeneity across bladder immune cells using enriched and optimized protocols.

Ligon et al. examined immune cell composition and the effects of aging on the bladder’s immune environment, using young and aged female mice as models [13]. Briefly, female mouse bladders were dissociated and subjected to a droplet-based scRNA-seq. Subsequently, urothelial and fibroblast cells were excluded from the analysis, focusing mainly on *CD45*^+^ cells (immune cells). The authors annotated 21 immune cell clusters, including macrophages, monocytes, dendritic cells, natural killer cells, and various subsets of B and T cells. Strikingly, both young and aged bladders contained two unique macrophages: a small subset distinguished by the expression of *Retnla*, and a large macrophage cluster. Additional subsets of macrophages that expressed the chemokine *Cxcl13* were upregulated solely in the aged bladder. Also present in the bladders were four subsets of dendritic cells, which included one cluster of type 1 conventional dendritic cells (cDCs) and three clusters of type 2 cDCs.

Interestingly, the analysis showed an age-dependent increase in B and T cell populations. B cells and T cells were either absent or present in a small fraction in the young bladder. The identified B cells were of various states: naïve, activated, and differentiated B cells. Other B cell clusters expressed *Fcrl5*, a marker of dysfunctional B cells, which was postulated to be involved in age-related inflammatory conditions and autoimmune disorders. Similarly, the aged bladder showed a remarkable increase in both CD4^+^ and CD8^+^ T cell subsets, as later verified by histological analysis. In contrast to the young bladder, histological sections of the aged bladder contained T cells and differentiated B cells which were aggregated in the lamina propria, forming structures of organized lymphoid tissues previously reported in models of chronic bacteriuria and bladder cancer. Functional studies also revealed that these organized structures helped recruit, activate, and differentiate B cells into IgA-secreting plasma cells with the support of proinflammatory cytokines.

In contrast, Baker et al. clustered immune cells into 13 distinct cell types: eight myeloid and five lymphoid subtypes [11]. The dataset was further integrated into a publicly available mouse PBMC dataset to identify potential resident immune cells in the bladder. The integrated clustering revealed two tissue-resident myeloid populations in the bladder: conventional dendritic cell 2 (cDC2) and macrophages (*Adgre1*^+^*)*. Interestingly, spatial transcriptomics revealed regional specificity for the subtypes: *Adgre1*^+^ macrophages (interstitial macrophages previously found in the lung), MHCII^+^ monocytes, and cDC1 were more likely to reside in the detrusor while the resident cDC2 (*Xcr1*^+^) were localized to the urothelium/lamina propria. Like the findings from Ligon et al., the spatial representation from Baker et al. also uncovered the presence of tertiary lymphoid structures in aged bladder sections. An increased number of spots denoted by the plasma cell marker *Jchain* were detected in Visium sections (no. of spots: Aged = 462 vs. Young = 3).

**Fig. 3:**
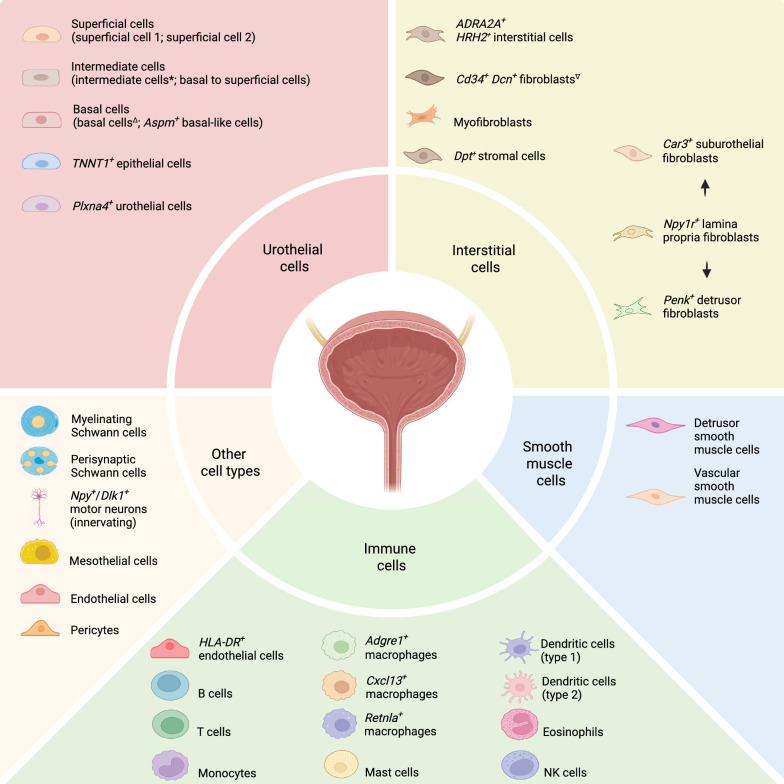
Cell composition of the urinary bladder identified by sc/snRNA-seq. Major cell types are grouped into five categories: urothelial, interstitial, smooth muscle, immune, and other cell types. Several subpopulations of cells exist, as listed under each category and in the main text. *Subtypes of intermediate cells include intermediate cells 1 and intermediate cells 2. Similarly, ∆basal cells are subdivided into basal cells 1, basal cells 2, etc. ∇Other names exist for *Cd34*^+^*Dcn*^+^ fibroblasts (e.g., FibSmo). *Npy1r*^+^ lamina propria fibroblasts can transdifferentiate into *Car3*^+^ suburothelial or *Penk*^+^ detrusor fibroblasts. Created in https://BioRender.com

Collectively, both Ligon et al. and Baker et al. present known and novel immune cell populations in the urinary bladder whose compositions are dependent on the age of the subjects [11, 13]. The two studies highlight that the aged bladder is densely populated with plasma cells and that the urinary bladder contains several resident immune cells, including *Adgre1*^+^ macrophages and cDC2, among others (Fig. 3). The relevance of these studies in human subjects requires further exploration to dissect the immune landscape and its role in bladder physiology.

### Minor cell populations: endothelial cells, mural cells, mesothelial cells, and neurons

The urinary bladder also contains other cell types, including endothelial cells, mural cells (pericytes and vascular smooth muscle cells), mesothelial cells, and neurons. These cells represent a small fraction of scRNA-seq datasets. Capturing the heterogeneity across these cells remains challenging, possibly because their composition is too minute to resolve by clustering analysis or detect through the currently available methods. We devote this section to present evidence of these cell types from scRNA-seq studies.

### Endothelial cells

Endothelial cells typically express genes found in neighboring tissues and, as such, are clustered close to vascular smooth muscle cells and fibroblasts [20]. Most scRNA-seq studies to date assign all endothelial clusters to the general markers of endothelial cells, including VIM, SELE, PECAM1 (CD31), VCAM1, and CDH5. Although all endothelial cells share several similarities, distinct genetic signatures of endothelial cells have been reported for organs such as lung, heart, and brain [20]. Unfortunately, finding bladder-specific endothelial cells remains limited. So far, one identified study has attempted to define endothelial cells of bladder origin. Han et al. characterized 14 major clusters of endothelial cells, of which one, specific to the bladder, expressed MHC class II genes such as *HLA-DPA1* and *HLA-DRA* [21] (Supplementary Table 2). Immunofluorescence staining for HLA-DR was found to colocalize with the classical endothelial marker, CD31 (PECAM1), in the urinary bladder. However, not all *CD31*^+^ endothelial cells stained positive for HLA-DR, which implied that *HLA-DR*^+^ endothelial cells are unique subsets. Moreover, the expression of antigen-presenting genes indicates a potential immune role of endothelial cells in the bladder. These results also suggest that CD31, a marker which is widely used to describe endothelial cells, is insufficient for defining tissue-specific endothelial cells.

### Mural cells

Mural cells constitute pericytes and vascular smooth muscle cells. The Muhl et al. analysis characterized and compared mural cells to fibroblasts originating from four muscular organs, including the urinary bladder [8]. While the identity of fibroblasts differed across and within organs, mural cells were more homogeneous and shared similar cells across multiple organs. A heat map of the top 50 marker genes revealed similar transcripts between pericytes and vascular smooth muscle cells across all four organs except for two clusters unique to the colon and heart. In addition, pericytes could transition into smooth muscle cells by expressing *Acta2*, *Myh11*, and *Rgs5*. Although mural cells did not express the fibroblast gene, *Pdgfra,* no universal marker defined all mural cells. Pericytes were therefore characterized by the expression of *Rgs5.* In stark contrast, vascular smooth muscle cells expressed contractile proteins including *α-SMA* and *Acta2* (Supplementary Table 2). While vascular smooth muscle cells represented a large fraction of mural cells, as few as 10 pericytes were identified in the bladder. The authors attributed this low cell capture rate to their sensitivity and poor survival during dissociation. They are also visibly present in capillaries and could adhere tightly to endothelial cells or venous smooth muscle cells, making their separation from endothelial cells nearly impossible.

### Mesothelial cells

Adjacent to the detrusor and outermost of the bladder wall reside monolayered cells known as mesothelial cells. These specialized cells provide a non-abrasive surface allowing the bladder to expand and contract with reduced friction to other surrounding tissues [22]. Little is known about their diverse roles in the bladder, let alone their properties and individual types (if there are any). Mesothelial cells were identified as one of the clusters in the Baker et al. study [11]. The authors described a cluster of cells which highly expressed the mesothelial gene *Msln* and the glycoprotein *Gpm6A*. A similar group of cells expressing *Gpm6A* was identified in the mouse datasets from Yu et al. [6]. However, Baker et al. argued that Yu et al. had wrongly annotated the cell cluster as “neurons”, as these cells also expressed *Upk3b, Acta2, Krt19*,* and Vim.*

### Neurons and neuronal moieties

Few studies have annotated sporadic clusters of neurons in the urinary bladder. The bladder is known to lack neuronal bodies, but it is well innervated with the autonomic nervous system interacting with several cells, including the smooth muscle cells, for contraction. Capturing these neuronal moieties in single-cell resolution presents challenges as neurons are sensitive to dissociation and die as a result [23]. However, snRNA-seq offers an alternative approach to preserving and capturing neurons. This is particularly evident in the preprint authored by Baker et al., wherein a combination of snRNA-seq and spatial transcriptomics allowed for the characterization of neuronal processes in the bladder [11]. Positive regions in the spatial transcriptomic data revealed the expression of two key neuronal-specific transcripts: *Npy* and *Slc17a7 *(VGLUT1), indicative of neuronal processes. *Npy*^+^ cells co-expressed *Dlk1*, a gene not only found in motor neurons but also in fibroblasts. Interestingly, an independent immunostaining of bladder sections with DLK1, PGP9.5, and CD34 revealed that *Dlk1* is co-expressed in both neurons innervating the bladder and the detrusor muscle fibroblasts.

Besides the *Npy*^+^ neural gene, other neuron-positive regions containing *Slc17a7*^+^ cells expressed the canonical gene marker *S100b*, which characterizes Schwann cells. This revelation prompted further sub-clustering of this group into two subtypes: myelinating and non-myelinating Schwann cells. Schwann cells have also been identified by other studies, including Santo et al., discussed in other sections [12]. These data collectively highlight the presence of neuronal moieties in the urinary bladder contributing to the diversity of cell types in the bladder.

### A map of bladder cells in relation to other cell types

Individual cells are a part of a continuous biological system working in harmony to support the organism. As a result, similar cells could be present in multiple tissues presenting parallel or distinct functions in a multi-organ context. Multi-organ analysis aims to map individual cells across several organs, providing patterns and interactions between the cells at various anatomical locations. Here, we review five studies that presented a global cell atlas of multiple organs, including the bladder, with an attempt to find relationships between the cells of the bladder and those of other organs.

Han et al. profiled over 50 different mouse organs, tissues, and cell lines using a microwell-based scRNA-seq [24]. Of the individual cells analyzed, 3143 cells were transcriptionally unique to the bladder. Cell clusters of the bladder included vascular smooth muscle progenitor cells, NK cells, endothelial cells, dendritic cells, macrophages, epithelial cells, umbrella cells, vascular endothelial cells, and smooth muscle cells. Furthermore, a cross-tissue analysis was performed to identify whether tissue-specific subtypes existed among these major cell types and to investigate their relationship within and across the organs. The authors identified 21 clusters of tissue-specific stromal cells wherein two clusters were bladder-specific, both marked by the expression of *Bmp4* and *Wnt2* (Supplementary Table 2). One cluster had a high enrichment for the chemokine *Cxcl12* and the proliferation marker *Ifitm1*, while the other cluster expressed high levels of *Bmp5* and *Car3* in line with results published by Baker et al. and Muhl et al. [8, 11] (see section on interstitial cells). The authors further identified 13 subclasses of macrophages based on their expression of C-type lectin domain family proteins. Of the 13 subtypes, a cluster of tissue-resident macrophages was found in the bladder, indicating that tissue macrophages, as opposed to blood-related macrophages, are specialized to adapt to a specific organ’s micro niche. This cluster of macrophages was also identified in multiple organs including the testis, pancreas, and mammary gland.

Following their previous publication of the mouse cell atlas, Han et al. reconstructed a human cell atlas [21]. Applying the same technique as previously mentioned, the authors profiled 60 human tissues and cell cultures. Specifically, two adult bladder tissues were analyzed, resulting in the clustering of urothelial, stromal, and smooth muscle cells. Antigen-presenting endothelial cells were also present in the bladder, sharing similar transcripts as those in the kidney. The authors also identified the expression of immune-related genes in several other non-immune cells, including endothelial cells, interleukin-expressing stromal cells, and *CXCL*^+^ epithelial cells in the bladder and other organs. Such discoveries represent a paradigm shift in our understanding of tissue-resident immunity, especially concerning epithelial cells of the bladder, which are constantly exposed to toxins and pathogens.

In another study, He et al. systematically mapped individual cells from 15 different organs resected from a single adult donor [25]. The resulting human cell atlas generated a transcriptome of over 84,000 cells, which was grouped into 43 clusters of cells. Across multiple organs, the authors observed close clustering of cells for major cell types such as endothelial, fibroblasts, and smooth muscles, suggesting they shared a similar transcriptome beyond their microenvironment. Among the cells, 7572 bladder cells were segregated into distinct cell types based on previously known markers. A range of newly identified clusters, assigned as FibSmo, were predominantly identified in the bladder (17.59%); however, a small proportion was also found in the rectum (6.66%) and the heart (7.75%). These novel clusters representing fibroblasts and smooth muscle cells showed a high enrichment for *MMP2*, *DCN*, and *ACTA2*. Evidently, the co-expression of *ACTA2* and *MMP2* was detected in the submucosa of the bladder by an immunofluorescence assay. Additional confirmation of this fibroblast cluster was supported by other pre-existing single-cell datasets from Yu et al. and Han et al., revealing similar co-expression of *MMP2, ACTA2*, as well as *PLAT*, *BMP4*, and *BMP5* (Supplementary Table 2).

One advantage of performing multi-organ analysis is that it allows for a comparative analysis of various cell types across multiple tissues. Comparing the datasets with the previous human cell atlas from Han et al., the authors from the He et al. study revealed that the epithelial cells of the bladder clustered more closely to the epithelial cells of non-digestive organs, including the trachea, ureter, and prostate. In fact, epithelial cells of non-digestive tissues shared similar transcription factors (TFs) as those of the bladder. This finding also echoes that TFs specify cellular identity and thus define tissue heterogeneity across tissues like epithelium that share common lineages and functions.

The final multi-organ studies to be discussed are two studies from the Tabula Muris Consortium [26, 27]. One study pertains to the cell atlas of the mouse, the *Tabula Muris*, while the other presents a cell atlas across the lifespan of a mouse, termed *Tabula Muris Senis*. In the *Tabula Muris*, the consortium analyzed single cells from three female and four male mice comprising 20 organs, including the urinary bladder [26]. The authors mechanically dissected the bladder mucosa and stroma from the detrusor and processed the stripped epithelial/stroma sheet on two single-cell platforms: one approach was a single-cell-sorted fluorescence-activated cell sorting (FACS)-based, which offers a full-length transcript analysis and provides a high gene coverage; the other method, a conventional microfluidic droplet-based 3’-end transcript, which captures many cells at relatively low coverage. Both platforms revealed a similar number of urothelial and mesenchymal cells (fibroblasts). However, when using the FACS method, cells are preselected and sorted based on a molecular marker of interest (e.g., EPCAM), and as a result, rare and minor cell populations could be lost in the analysis. For example, *Pecam1*^+^ endothelial cells and *Cd14*^+^ immune cells were not captured in the FACS-bladder datasets as compared to those from the microfluidic platform. Nevertheless, both methods identified three urothelial cell populations: umbrella, intermediate, and basal cells (Fig. 3). Moreover, *Dcn*^+^ mesenchymal cells were named bladder cells and grouped into three subclusters, two of which were enriched in *Car3*, while the other was enriched in *Scara5* (Supplementary Table 2). Remarkably, these clusters are transcriptionally akin to the stromal cells identified by Baker et al. and Han et al. [11, 24].

The *Tabula Muris* captures gene expression profiles of mouse tissues; however, this cellular landscape represents only a snapshot of a young mouse. In their second publication, the authors investigated how cellular composition changed over the life span of the mouse [27]. Using mouse tissues spanning from 1 to 30 months, the consortium reported that age was associated with a disproportion of cells exemplified by both changes in the number of cells and gene expression. Older mice (30 months) had more cells that expressed *p16*, which plays a key role in senescence. Other genes including *E2f2*, *Lmnb1*, *Tnf,* and *Itgax* also correlated with aging. In contrast, fewer cells expressed genes that encode sirtuin proteins, which aligns with evidence pointing that sirtuins are essential for impeding cellular senescence at old age [28]. While controlling for sex and technical bias, the authors also reported that cellular composition differed with age across multiple tissues, including the bladder, liver, and kidney. In the bladder, mesenchymal cells decreased three times with age while urothelial cells increased in reciprocate as the mouse aged. Stromal-associated genes (e.g., *Col1a1*, *Col1a2*, *Col3a1*, and *Dcn*) were downregulated. In stark contrast, epithelial-associated genes (e.g., *Krt15*, *Krt18*, and *Sfn*) were upregulated with age. A similar trend was also seen in other cell types, such as endothelial cells, which had lower expression of vasculature-associated genes (e.g., *Htra1* and *Fos*), an indication that the aged bladder is poorly vascularized. Furthermore, the consortium also reported an increase in the number of leukocytes, which is indicative of age-related inflammation, echoing results from Ligon et al. [13].

Taken together, multi-organ analysis represents a global view of individual cells from several organs and tissues of an organism. While intra- and inter-organ differences exist, the bladder shares related cell types with different organs and, as such, multi-organ representations underscore how interconnected bladder cells are.

### A head-to-head comparison of scRNA-seq and snRNA-seq with urinary bladder tissues

A recent study by Santo et al. presented a head-to-head comparison between scRNA-seq and snRNA-seq [12]. The team sequenced and analyzed paired samples from four regions of the urinary bladder, including the dome, neck, ureteral orifice, and ureterovesical junction. The clustering analysis yielded 14 clusters representing three major cell types of the bladder: urothelial, stromal, and immune cells. Classification of each individual major cell type was performed to decipher their distinctive subtypes. For example, two unique smooth muscle clusters were identified as general smooth muscle cells and vascular smooth muscle cells from the stromal population (Supplementary Table 2). Other cell types identified included Schwann cells, arterial, and lymphatic cells, which appeared distinct from endothelial and other stromal cell gene signatures.

Besides cell identification, the study highlighted several key takeaways from both technologies. First, the authors found a significant disparity between the cell distribution across the two technologies as each technology favored the detection of a particular cell type. For example, intermediate cells were dominated by transcripts from single nuclei, while the basal and umbrella cells were prominent in the single-cell data. Likewise, single nuclei could provide a better resolution for capturing myofibroblasts, smooth muscle cells, and Schwann cells in line with results from Baker et al. [11]. Secondly, non-coding RNAs dominated the top differentially expressed genes (DEGs) of the single nuclei datasets, whereas the top DEG markers of the single cell were made up of canonical genes (e.g., *UPK2* or *KRT20* for urothelial cells; and *DES* or *MYH11* for smooth muscle cells). The presence of non-coding RNAs, represented in each cluster (e.g., intermediate cells), poses a challenge for deciphering cell clusters as previous cell identification efforts have been based on established markers originating from single cell-based data. Equally challenging, non-coding RNAs are poorly described for cell identification. However, this challenge highlights the significance of using non-coding RNA enriched single nuclei to identify rare cell populations, which could further our understanding of cellular diversity. Third, the evaluation of biological processes using gene set enrichment analysis also revealed some differences between the two technologies. The top DEGs from snRNA-seq, particularly for urothelial and stromal cells, had more generic biological processes as compared to the scRNA-seq data, making it impossible to discriminate cell types with gene ontology. In contrast, identifying immune cell types with biological processes showed no difference between the two technologies.

## Discussion

This review provides a comprehensive overview of sc/snRNA-seq studies of the healthy urinary bladder in human, mouse, and rat. These 12 studies present the molecular profiling of cells found at various regions of the urinary bladder, with some studies tackling the challenges associated with isolating difficult-to-dissociate cell types. Although characterization of these cells has yielded similar reports across studies, an integrated approach is still needed to fully capture patterns across studies and modalities, as discussed below. We have attempted to summarize the top gene expression profiles of key cell types, with the aim of consolidating the results from these studies (Supplementary Table 2). For example, multiple studies, albeit with different naming of the cell clusters, confirmed the presence of *CD34*^+^ fibroblasts, which reside just below the urothelium. Discrepancies were also highlighted, such as the presence of detrusor smooth muscle cells and neurons, which were captured in studies that utilized the snRNA-seq approach over the scRNA-seq. These transcriptomic studies offer an untapped resource for therapeutic application in precision medicine within the realms of bladder cancer and benign bladder diseases. However, the application of single-cell technologies in urology must overcome the following challenges, among others.

Access to healthy human samples is still constrained. Currently, high-throughput profiling of the bladder is restricted to malignant conditions, and performing biopsies from benign bladder diseases is not a routine medical practice. Even when a biopsy is collected from a benign condition, such a limited tissue sample may not be representative of the bladder given the regional differences in the composition of fibroblasts and immune cells, as discussed in earlier sections. Studies have also shown structural and functional differences between the anatomical regions of the bladder dome and the trigone [9, 29, 30].

Although animal models and primary cells could compensate for the limited availability of human tissues, they may not fully replicate or represent the molecular intricacies of the human bladder. In fact, Yu et al. compared human and mouse bladders by investigating conserved and heterogeneous transcriptomic profiles of bladder cell populations [6]. The authors identified some shared characteristics, measured by a high Pearson correlation; however, subsets of fibroblasts and endothelial cells were dissimilar between species. Individual properties of urothelial cells, including the expression of cytokeratin genes, were also heterogeneous. Thus, the availability of human tissue is greatly desirable for our understanding of the complex molecular composition of the bladder.

The choice of single-cell profiling methods depends on tissue storage and preparation. Conventional single-cell methods require immediate dissociation of fresh tissues, which is time-consuming and impractical in the clinic. Conversely, snRNA-seq can use fresh frozen samples, decoupling tissue acquisition from the technological workflow and providing alternative measures to overcome the logistical challenge. Formalin-fixed paraffin-embedded (FFPE) samples, commonly used in pathology, offer additional benefits but pose challenges due to fragmented RNAs, making poly(A)^+^ RNAs capture difficult [31]. However, new methods are being developed to better analyze FFPE samples, and several have shown promising results compared to traditional scRNA-seq [31, 32].

Fresh frozen and FFPE-based snRNA-seq offer convenient alternatives, but gene expression and cluster identification can vary by method and tissue type. Each approach highlights a separate perspective of different cellular compartments. Santo et al. reported that intermediate cells from snRNA-seq were enriched in non-coding RNAs while scRNA-seq captured more overall transcripts and canonical markers [12]. Likewise, studies have reported greater cell diversity and non-coding RNA detection with snRNA-seq, also from FFPE samples [31, 32]. In addition, fresh-frozen/FFPE-based snRNA-seq avoids dissociation-induced stress response genes, an artifact associated with scRNA-seq [12, 33]. Earlier in this review, we highlighted that the detection of heterogeneity within detrusor smooth muscle cells and other cells is higher with snRNA-seq. Thus, sc/snRNA-seq technologies offer a partial view and as a result complementary studies to investigate chromatin accessibility, DNA methylation, metabolomics, etc., are essential for a complete understanding of urinary bladder physiology.

### Future directions

The development of technologies to elucidate cellular compartments will continue to accelerate in parallel with large volumes of generated data. We anticipate that large-scale integration of studies across various modalities will provide a holistic perspective. Given the challenges associated with sample availability, tissue preparation, and single-cell method, having a large cohort of human and animal studies is crucial, offering additional insights into sample variability. In addition, large-scale data analysis will provide a unique window to simplify transcriptomic analysis and to develop additional models while consolidating heterogeneity across multiple single-cell technologies and platforms. As Heumos et al. state: “multimodal reference atlases will further enable the characterization of cell states on several layers” [10]. This is equally true for a few studies that have already integrated multiple scRNA-seq studies of the urinary bladder.

By collecting published scRNA-seq datasets of female and male bladders, Wu et al. identified sex-based differences in gene expression profiles associated with the onset of urinary tract infection (UTI) and bladder cancer [14]. While further research is needed to fully comprehend the epidemiological disparities in UTI and bladder cancer, the study concluded that bladder tumors in females underscore a biologically distinct disease with potentially different outcomes. Likewise, through large dataset integration, Shi et al. uncovered significant insights into the diversity of bladder cells and their intercellular signaling communication, which could provide therapeutic benefits [34]. Thus, combining multiple scRNA-seq studies allows for identifying new patterns across a variable large cohort and providing new insights into treatments of bladder conditions.

Integration of large datasets is not limited to multiple scRNA-seq datasets and may also involve the analysis of multiple omics data types including metabolomics, epigenomics, miRNA, and many others, largely unexplored in urology. The urinary bladder is constantly exposed to pathogens, which may cause UTIs. Applying single-cell technologies to explore the complex microbiome of the urinary bladder could unlock new, currently untapped opportunities for targeted therapeutic approaches [35]. While all included studies but one analyzed the adult bladder, we have identified that knowledge of the cellular composition of the juvenile and regenerative urinary bladder remains limited, despite its relevance in the treatment of congenital genitourinary malformations. Harnessing specific subtypes of basal cells and suburothelial fibroblasts is paramount for improved bladder regeneration after bladder reconstruction and for future tissue engineering technologies. Nonetheless, we anticipate that future studies and ongoing initiatives through various consortia including the Human Cell Atlas, GenitoUrinary Development Molecular Anatomy Project (GUDMAP) and ReBuilding A Kidney (RBK), will drive these heavy data-driven studies [23, 36, 37].

### Strengths and limitations

We have applied a systematic search strategy to identify relevant studies that align with the research question and objectives. Limiting the language to English may have forced us to omit some relevant studies. However, due to the paucity of data in this field, the included studies represent the majority. In addition, we have included a non-peer-reviewed study, currently in preprint, offering unique and valuable data. It is therefore essential for the reader to interpret the methods and results from the preprint study with extra critical thought.

## Conclusion

The aim of the scoping review was to consolidate existing evidence on the use of sc/snRNA-seq to assess cellular diversity in the healthy urinary bladder, and to further identify gaps in the literature that could guide future research in this field. We have reported that the urinary bladder is heterogeneous, with multiple subsets of cells described for a given cell population located at various regions of the bladder. Although few studies exist on the healthy bladder, the results underline the complex molecular profile of the urinary bladder. Additional in-depth analysis integrating these results into a large dataset is critical for improved understanding of how these cells contribute to normal functions and diseases.

## Supplementary Information

Supplementary material 1. Search query.

Supplementary material 2. Summary of key clusters.

## Data Availability

All data generated are included in the article and its supplementary information files.

## Abbreviations

scRNA-seq: Single-cell RNA sequencing
sc/snRNA-seq: Single-cell or single-nuclei RNA sequencing
PRISMA: Preferred reporting items for systematic reviews and meta-analyses
ICs: Interstitial cells
ECM: Extracellular matrix
VIM: Vimentin
Plxna4: Plexin A4
suF: Suburothelial fibroblasts
lpF: Lamina propria fibroblasts
dmF: Detrusor muscle fibroblasts
cDCs: Conventional dendritic cells
FACS: Fluorescence-activated cell sorting
DEGs: Differentially expressed genes
FFPE: Formalin-fixed paraffin-embedded
UTI: Urinary tract infection
GUDMAP: GenitoUrinary Development Molecular Anatomy Project
RBK: ReBuilding a Kidney
