# Supplementary material for: Single-cell profiling defines the cellular landscape of the urinary bladder: a scoping review
Source: Eur J Med Res. 2026 Jan 5;31:192. doi: 10.1186/s40001-025-03750-6 (PMC12870175; doi:10.1186/s40001-025-03750-6)
Supplement: Supplementary file 1 — Supplementary material 1. Search query. [file 40001_2025_3750_MOESM1_ESM.docx]

| **Embase <1974 to 2024 September 03>** | | | |
| --- | --- | --- | --- |
| **Date of last search** | **Query** | | **Results** |
| Feb 17, 2025 | #1 | ("Gene Expression Profiling" or "Single-Cell Analysis" or "Transcriptome" or "RNA Sequence Analysis" or "Single-Cell Gene Expression Analysis" or "RNA analysis" or "Differentially Expressed Gene*" or "Single-Cell*" or "Single-cell RNA sequencing").af. | 523486 |
|  | #2 | (("urinary bladder" or "urothelium") not ("cancer" or "carcinoma")).af. | 24959 |
|  | #3 | #1 AND #2 | 231 |

| **PubMed (MEDLINE)** | | |
| --- | --- | --- |
| **Date of last search** | **Query** | **Results** |
| Feb 17, 2025 | (( ((((((((("Gene Expression Profiling"[Mesh]) OR "Single-Cell Analysis"[Mesh])) OR "Transcriptome"[Mesh]) OR "Sequence Analysis, RNA"[Mesh]) OR "Single-Cell Gene Expression Analysis"[Mesh]) OR "RNA/analysis"[Mesh])) OR (differentially expressed gene*[Text Word])) OR (single-cell*[Text Word]) ) OR (single-cell RNA sequencing[Text Word])) AND (((("Urinary Bladder"[Mesh]) OR "Urothelium"[Mesh]) NOT (cancer*[Title])) NOT (carcinoma*[Title])) | 444 |

| **Scopus** | | |
| --- | --- | --- |
| **Date of last search** | **Query** | **Results** |
| Feb 17, 2025 | ( TITLE-ABS-KEY ( "Gene Expression Profiling" OR "Single-Cell Analysis" OR "Transcriptome" OR "RNA Sequence Analysis" OR "Single-Cell Gene Expression Analysis" OR "RNA analysis" OR "differentially expressed gene*" OR "single-cell*" OR "single-cell RNA sequencing" ) AND TITLE-ABS-KEY ( "Urinary Bladder" OR "Urothelium" ) AND NOT TITLE-ABS-KEY ( "cancer*" OR "carcinoma*" ) ) | 375 |

| **Web of Science** | | | |
| --- | --- | --- | --- |
| **Date of last search** | **Query** | | **Results** |
| Feb 17, 2025 | #1 | ((((((((TS=(Gene Expression Profiling)) OR TS=(Single-Cell Analysis)) OR TS=(Transcriptome)) OR TS=(RNA Sequence Analysis)) OR TS=(Single-Cell Gene Expression Analysis)) OR TS=(RNA analysis)) OR TS=(Differentially expressed gene*)) OR TS=(Single-cell*)) OR TS=(Single-cell RNA sequencing) | 816,748 |
|  | #2 | ((((TS=(urinary bladder)) OR TS=(urethra)) OR TS=(urothelium)) NOT TS=(cancer*)) NOT TS=(carcinoma*) | 63,447 |
|  | #3 | #1 AND #2 | 533 |

Google search:

- “Single-cell/Single-nuclei RNA sequencing and urinary bladder”
- “Urinary bladder and single cell”
