# Supplementary material for: Single-cell profiling defines the cellular landscape of the urinary bladder: a scoping review
Source: Eur J Med Res. 2026 Jan 5;31:192. doi: 10.1186/s40001-025-03750-6 (PMC12870175; doi:10.1186/s40001-025-03750-6)
Supplement: Supplementary file 2 — Supplementary material 2. Summary of key clusters. [file 40001_2025_3750_MOESM2_ESM.docx]

Supplementary Table 2. Summary of key clusters

| Reference and  source of data | Species | Gene signature | Data repository |
| --- | --- | --- | --- |
| Yu et al. 2019 (6)  Supplementary Table 9 and main text | Human | ***ADRA2A^+^ HRH2^+^* interstitial cells:** *MALAT1****,*** *TAGLN, S100A6, RPL41, RPS27, ACTA2, AVPR1A, HRH2, RGS5, ADRA2A*  ***TNNT1^+^* epithelial cells:** *FTH1, RPL10, TMSB4X, RPS27, KRT18, KRT19, TNNT1, CST6, UPK2, UPK3B* | GSE129845 |
| Zhao et al. 2023 (17)  Supplementary Table S2 and Table S3 | Human  Rat | **Fibroblasts*:** *DCN*, *CFD*, *MGP*, *IGFBP6*, *GSN*, *FBLN1*, *DPT*, *PCOLCE2*, *APOD*, *TNFAIP6*  **Myofibroblasts:** *STC1*, *AREG*, *PLAT*, *AKR1C1*, *TSLP*, *TNC*, *TRPA1*, *TBX3*, *A2M*, *PDK4*  **Fibroblasts*:** *Pla2g2a*, *Mgp*, *Col3a1*, *Col1a1*, *Mfap5*, *Gsn*, *Tnfaip6*, *Sparc*, *Dcn*, *Fbn1*  **Myofibroblasts:** *Cxcl14*, *Tgfbi*, *Cryab*, *Car3*, *Stmn2*, *Igfbp4*, *Cyp1b1*, *Htra1*, *Col12a1*, *Vim* | GSE164557 |
| Li et al. 2021 (9)  Figs. 1B, 1D and 8 | Mouse | ***Plxna4^+^* cells:** *Anxa1, Col6a1, Ildr2, Lrrn4, Ltbp4, Muc16, Plnxna4, Sema3c, Trp53, Upk3b*  ***Aspm^+^* basal-like cells:** *Anxa1, Arid1a, Aspm, Ccna2, Cd44, Itgb4, Kif4, Krt5, Shh, Trp63* | GSE163029 |
| Baker et al. 2021 (11)  Main text; Fig. 3C and Supplementary Table S8 | Mouse | ***Car3^+^* suburothelial fibroblasts**^∆^**:** *Car3, Cxcl14, Gas6, Acta2, Rbp4, Fbln1, Thy1, Pi16*  ***Npy1r^+^* lamina propria fibroblasts:** *Npy1r, Pi16, Fbln1, Lum, Cd34*  ***Penk^+^* detrusor fibroblasts:** *Penk, Dpt, Pi16, Fbln1, Lum, Cd34, Sultle1, Dlk1*  **Myelinating Schwann cells:** *Igfbp6, Mpz, Pllp*  **Perisynaptic Schwann cells:** *Pdgfb, Scn7a, Prnp, Apoe*  **Vascular smooth muscle cells:** *Pln, Crip1, Mustn1, Tesc, Wtip*  **Detrusor smooth muscle cells:** *Actg2, Acta1, Tnnt2, Mylk, Cnn1, Synpo2*  **Pericytes:** *Rgs5, Colec11, Ifitm3, Pdgfrb, Kcnj8* | GSE180128 |
| Muhl et al. 2020 (8)  Supplementary Table 1, Supplementary Fig. S2, and Supplementary Table 3 | Mouse | ***Tnc^+^ Cd34^-^* fibroblasts:** *Adam23, Col6a3, Tgm2, Gata5, Tnc, Car3, Sfrp2, Dkk2, Col16a1, Bmp3*  ***Tnc^-^ Cd34^+^* fibroblasts:** *Cd34, Tcf21, Mgp, Dcn, Igfbp6, Scara5, Serpinf1, Sparc, Pi16, Gsn*  **Bladder mural cells:** *Kcne4, Ramp1, Btg2, Atf3, Cebpb, Efhd2, Cdk11b, Fosl2, Coro1c, Pdgfa* | GSE150294 |
| Ligon et al. 2020 (13)  Supplementary Fig. S3 and Supplementary Table S2 | Mouse | ***Retnla^hi^* macrophages:** *Retnla, Fcna, Folr2, Mafb, Cd209f, Lyve1, F13a1, Clec10a, Ccl24, C4b*  **Bladder macrophages:** *Mgl2, Sirpa, Adgre1, Ccl7, Ccl12, Ccl2, C1qa, Ccl8, C1qc, Pf4*  ***Cxcl13^+^* macrophages:** *Nr1h3, Colq, Cxcl13, Ccl8.2, Fabp5.1, C3, Gdf15, Cd63.3, C1qb.17, C1qa.17* | GSE149571 |
| Han et al. 2018 (24)  Supplementary Table S5 | Mouse | **Stromal cells (*Dpt^hi^*):** *Dpt, Gas1, Cxcl12, Mgp, Clec3b, Mfap4, Htra3, Serpinf1, Gsn, Cd55*  **Stromal cells (*Car3^hi^*)** ^∆^**:** *Car3, Cxcl14, Tnc, Rbp4, Dkk2, Adamdec1, Bmp5, Thbs2, Aldh1a2, Gpx3*  **Tissue resident macrophages:** *Pf4, C1qc, C1qa, Apoe, Ccl8, Mrc1, Csf1r, Mgl2, Clec10a, Cd209f* | GSE108097 |
| Han et al. 2020 (21)  Supplementary Table 3 | Human | **Bladder-specific endothelial cells:**  *AQP1, IL6, PECAM1, HLA-DRB1, VWF, POSTN, IGFBP4, HLA-DRA, SPRY1, HLA-DPA1* | GSE134355 |
| He et al. 2020 (25)  Supplementary Fig. S23 | Human | **FibSmo cells:** *MMP2, DCN, PLAT, ID1, COL1A1, COL3A1, BMP4, BMP5, ACTA2, MYH11* | GSE159929 |
| The Tabula Muris Consortium 2018 (26)  Supplementary information | Mouse | **Droplet:**  **Bladder cell/mesenchymal cells**^∆^**:** *Car3, Col1a1, Col8a1, Dcn, Scara5*  **Bladder urothelial cells:** *Epcam, Grhl3, Krt5, Krt14, Upk1b, Upk3a*  **Endothelial cells**: *Pecam1*  **Leukocytes:** *Cd14, Ptprc*  **FACS:**  **Bladder cells**^∆^**:** *Car3, Col1a1, Col8a1, Dcn, Scara5*  **Bladder urothelial cells:** *Cd14, Epcam, Grhl3, Krt5, Krt14, Upk1b, Upk3a* | GSE109774 |
| The Tabula Muris Consortium 2020 (27)  Fig. 2F and  Supplementary Table 6 | Mouse | **Downregulated stromal-associated genes (bladder cell genes)** ^∆^**:** *Car3, Col1a1, Col3a1, Col5a2, Col6a2, Dcn, Htra1, Mgp, Serpinh1, Sparc*  **Downregulated bladder endothelial cells:** *Zfp955b, Gm2027, Txnip, Sparc, Col3a1, Sdpr, Tsc22d3, Agbl5, Col1a2, Fam198b,*  **Upregulated epithelial-associated genes (urothelial cell genes):** *Ly6d, Gsta4, Krt18, Sfn, Krt8, Areg, Igfbp2, Gstm1, Cldn4, Krt7*  **Upregulated bladder leukocytes:** *Ccl5, Srgn, Gm11428, Lgals3, Rbp4, Cstb, Rps24, Ccl8, Clec7a, Cd44* | GSE132042 |
| Santo et al. 2025 (12)  Supplementary Table S3 and S4 | Human | **scRNA-seq**  **Smooth muscle cells:** *ACTC1, RBFOX3, DES, PCP4, ACTG2, AC079313.2, KCNQ5, P2RX1, NCKAP1L, PTGS1*  **Vascular smooth muscle cells:** *RGS5, GPR20, LRRC10B, KCNAB1, FHL5, RERGL, CDH6, NDUFA4L2, ADRA2A, NOTCH3*  **snRNA-seq**  **Smooth muscle cells:** *ASB5, RBFOX3, GTSF1, AC233701.1, PCA3, CHRM3-AS2, COL19A1, KCNN2, AC005180.1, AC079313.2*  **Vascular smooth muscle cells:** *FHL5, AC013565.1, GPR20, SLC6A1, AL450332.1, NMNAT2, ADRA2A, RGS6, NOTCH3, ANO3* | GSE267964 |

*The gene expression of fibroblasts was comparable between humans and rats.

∆Similar cell types were identified across multiple studies through data integration.
